# High-Depth RNA-Seq Data Sets for Studying Gene Expression Changes Mediated by Phase-Variable DNA Methyltransferases in Nontypeable Haemophilus influenzae

**DOI:** 10.1128/MRA.01500-18

**Published:** 2019-01-10

**Authors:** John M. Atack, Lauren O. Bakaletz, Michael P. Jennings

**Affiliations:** a Institute for Glycomics, Griffith University, Gold Coast, Queensland, Australia; b Center for Microbial Pathogenesis, The Research Institute at Nationwide Children’s Hospital and The Ohio State University College of Medicine, Columbus, Ohio, USA; University of Rochester School of Medicine and Dentistry

## Abstract

Nontypeable Haemophilus influenzae (NTHi) is a major bacterial pathogen that causes multiple infections. We report high-depth-coverage RNA-Seq data from three NTHi strains, each of which encodes a different phase-variable methyltransferase. Major gene expression differences occur, commensurate with *modA* phase variation, and data will serve as an important resource for future studies.

## Abstract (precis)

Nontypeable Haemophilus influenzae (NTHi) is a major bacterial pathogen that causes multiple infections. We report high-depth-coverage RNA-Seq data from three NTHi strains, each of which encodes a different phase-variable methyltransferase.

## ANNOUNCEMENT

Nontypeable Haemophilus influenzae (NTHi) is responsible for human respiratory tract infections and invasive disease (1–3). Previous work characterizing NTHi showed that phase-variable N^6^-adenine DNA methyltransferases (ModA) are involved in epigenetic regulation and virulence (4–7). Phase-variable methyltransferase expression leads to genome-wide methylation differences, epigenetically regulating multiple genes, namely, a phasevarion (phase-variable regulon) (8, 9). *modA* alleles show high variability (<25% nucleotide identity) in their central, target recognition domain (TRD) that dictates specificity (10). Different TRDs methylate different sequences and define a phasevarion (8). We have shown that ∼65% of otitis media clinical isolates possessed one of five *modA* alleles, *modA2*, *4*, *5*, *9*, and *10* (5). We used NTHi strains C486 (*modA4*), 477 (*modA5*), and 1209 (*modA9*) (5) and generated variants where we locked each *modA* allele on (expressed) or off (not expressed) by reducing the number of AGCC_[n]_ repeats so that genes could not phase-vary using standard genetic techniques. Using these strains, we prepared triplicate biological replicates of total RNA with TRIzol reagent (Thermo Fisher) according to the manufacturer’s instructions from mid-log cultures grown in brain-heart infusion (BHI; Oxoid, UK) broth at 37°C with 150 rpm shaking in an aerobic atmosphere.

Libraries were prepared using the Illumina Ribo-Zero Gold protocol and assessed using an Agilent Bioanalyzer DNA 1000 chip. qPCR quantification was used to assess individual libraries before normalizing (2 nM) and pooling using the Illumina cBot system with TruSeq PE Cluster kit v3 reagents. Sequencing (150 bp paired-end runs) was performed on the Illumina NovaSeq system with TruSeq SBS kit v3 reagents. The average number of sequence reads for each triplicate sample is as follows: *modA4* on, 56,173,140; *modA4* off, 53,157,713; *modA5* on, 50,496,014; *modA5* off, 53,084,278; *modA9* on, 59,341,255; *modA9* off, 57,150,972). Sequence quality was assessed according to the standard protocols of the Australian Genome Research Facility (AGRF). Sequence reads were aligned against the respective reference genomes (GenBank accession numbers CP007471 [C486; *modA4*], CP007470 [477; *modA5*], and JMQP00000000 [1209; *modA9*]) with Bowtie 2 aligner (v2.3.3.1) using standard settings. Default software settings were used unless otherwise stated. Transcripts were assembled with StringTie v1.3.3 (http://ccb.jhu.edu/software/stringtie/) with the reads alignment and reference-annotation-based assembly option. This methodology generated assemblies for known and potentially novel transcripts. The GENCODE annotation that contained both the coding and noncoding annotation for each genome was used as a guide (http://www.gencodegenes.org/). Raw gene count values were analyzed with edgeR (https://bioconductor.org/packages/release/bioc/html/edgeR.html) to compute differential gene expression values. Counts were summarized at the gene level with the featureCounts v1.5.3 utility of the subread package (http://subread.sourceforge.net/). Gene expression differences between respective *modA* locked on and off were expressed as logFC (log_2_-fold change of expression). Our analysis generated logCPM values (average log count per million for the gene across all samples), *F* values (quasi-likelihood *F* statistic for the gene across all samples), *P* values for the test of a statistically different expression, and the FDR (false discovery rate/adjusted *P* value for multiple hypothesis testing).

By setting a cutoff for a greater than 2-fold differential expression, and excluding rRNA and tRNA genes, we show that 55 genes were downregulated and 82 genes were upregulated in strain C486 when *modA4* was on, 74 genes were downregulated and 14 genes were upregulated in strain 477 when *modA5* was on, and 116 genes were downregulated and 76 were upregulated in strain 1209 when *modA9* was on. Differentially regulated genes include those involved in central metabolism, nutrient acquisition, and stress response. We have previously identified the sites recognized by the ModA4, ModA5, and ModA9 methyltransferases (5). By combining this information with these new RNA-Seq data, we demonstrate that gene expression changes mediated by *modA* phase variation are complex and likely involve both the primary and secondary regulation of genes. These data will serve as a major resource for dissecting the exact molecular mechanisms of *modA*-mediated gene expression differences and for studying the cascade events that result in multiple gene expression differences commensurate with genome-wide methylation differences.

### Data availability.

The data announced here were deposited in GEO DataSets under series GSE121835 (full data set), GSE121832 (*modA4* on versus off), GSE121833 (*modA5* on versus off), and GSE121834 (*modA9* on versus off).
